# Advancing the Science of Mentorship: Future Directions for Sustainable Implementation and Evaluation of Mentorship Education for the Clinical and Translational Science Workforce

**DOI:** 10.1017/cts.2024.497

**Published:** 2024-03-13

**Authors:** Pam Asquith, Melissa McDaniels, Adriana Baez, Leonor Corsino, Roger Fillingim, Doris Rubio, Christopher Russell, Christine Sorkness, Winston Thompson, Christine Pfund

**Affiliations:** 1 Institute for Clinical and Translational Research, University of Wisconsin-Madison, Madison, WI, USA; 2 Center for the Improvement of Mentored Experience in Research, University of Wisconsin-Madison, Madison, WI, USA; 3 Departments of Pharmacology and Otolaryngology, School of Medicine, University of Puerto Rico, San Juan, PR, USA; 4 Division of Endocrinology, Metabolism and Nutrition and Population Health Sciences, Duke University School of Medicine, Durham, NC, USA; 5 College of Dentistry and Pain Research and Intervention Center of Excellence, University of Florida, Gainesville, FL, USA; 6 Institute for Clinical Research Education, Schools of the Health Sciences, University of Pittsburgh, Pittsburgh, PA, USA; 7 Department of Clinical Pediatrics, Keck School of Medicine, University of Southern California and Division of Hospital Medicine, Children’s Hospital Los Angeles, Los Angeles, CA, USA; 8 Departments of Physiology and Obstetrics and Gynecology, Morehouse School of Medicine, Atlanta, GA, USA

**Keywords:** mentorship, workforce development, CTSA, diversity, community of practice

## Abstract

The Advancing the Science of Mentorship: Future Directions for Sustainable Implementation and Evaluation of Mentorship Education for the Clinical and Translational Science Workforce conference was held in Madison, Wisconsin, in April 2023. The conference provided an engaging and scholarly forum for clinical and translational researchers from diverse backgrounds and career stages (including leaders at Clinical and Translational Science Award (CTSA) hubs and affiliated institutions) with a professional interest and commitment to improving and diversifying workforce development and fostering a climate of inclusive excellence through best practices in mentorship. Outcomes from the conference include an online resource and a new Community of Practice.

## Introduction

Effective mentorship is critical to the professional development of those engaged in biomedical research careers, including clinical and translational investigators. The Clinical and Translational Science Awards (CTSA) Program has recognized that advancing mentorship (including interventions to increase access to and the quality of mentoring relationships) is key to fostering the growth of a diverse clinical and translational research workforce [1–4]. The overall objective of the conference “Advancing the Science of Mentorship: Future Directions for Sustainable Implementation and Evaluation of Mentorship Education for the Clinical and Translational Science Workforce” was to build upon the contributions to mentorship education and the science of mentorship from many CTSA hubs [1–7]. The conference session themes were anchored in the key findings and recommendations from the National Academies Report on the Science of Effective Mentorship in STEMM [8].

## Identity as a mentor is key to diversifying the clinical translational workforce

Vivian Lewis, MD, is a Professor Emerita in the Department of Obstetrics and Gynecology at the University of Rochester where she previously served as the Vice Provost for Faculty Development and Diversity and Chair of the Mentor Development Group in the Clinical Translational Science Institute. Dr Lewis’s areas of professional interest include mentoring, leadership coaching, equity, diversity, and inclusion in healthcare. Dr Lewis delivered the opening plenary session: “Identity as a Mentor is Key to Diversifying the Clinical Translational Workforce.” Dr Lewis drew on the 2023 NASEM report, *Advancing Antiracism, Diversity, Equity* and Inclusion in STEMM Organizations: Beyond Broadening Participation [9] to illustrate the critical role that both sociocultural and mentor identities play in advancing anti-racism, diversity, equity, and inclusion in STEMM organizations and discussed research [10] on how mentor identity evolves and is shaped by personal and professional contexts. Dr Lewis highlighted the merits of the Executive Leadership in Academic Medicine (ELAM®), a leadership development program, where senior women leaders in academic health sciences engage in project work that shapes their leadership identities [11] She highlighted how mentor identities are necessarily connected to social, professional, and individual identities and are impacted by organizational and institutional contexts (e.g., CTSA hub, department, and institution). Importantly, Dr Lewis challenged attendees to use the conference as an opportunity to reflect and learn more about diversity, equity and inclusion (DEI) in order to both deepen their mentoring practice and promote the integration of sociocultural identity awareness into their own mentoring identity. Participants were encouraged to create action plans to promote DEI in their own settings as mentors and leaders.

## Responsive mentorship promotes trainee resilience

Emma Meagher, MD, is a Professor of Medicine and Pharmacology, and Director of Translational Research Education at the Perelman School of Medicine at the University of Pennsylvania. Dr Meagher’s presentation “Responsive Mentoring for Uncertain Times” explored the important roles that both mentors and mentees play in optimizing mentoring relationships, with a specific focus on the development of mentoring skills that enhance trainees’ resilience. Reflecting on the immediate impact of COVID-19 on trainees’ career development [12], Dr Meagher discussed the importance of balance between individual (e.g., clarity of goals, accountability, and resilience) and environmental factors (e.g., developmental frameworks, institutional culture, and mentorship) in promoting mentor and mentee responsiveness. She described a responsive mentor as one who: 1) cultivates belonging to the professional community, 2) builds and reinforces mentees’ self-efficacy, 3) is skilled at delivering critical feedback, and 4) builds trust through thoughtful communication. Dr Meagher emphasized the critical role that mentees must play in navigating competing demands from four domains: work, home, self, and community. The four-way view assessment developed and published by Stewart Friedman [13] was shared as a tool that mentees can use to engage with others to 1) clarify what is important to them as they plan future goals and 2) articulate a personal leadership vision in the years to come. This self-assessment was incorporated into a structured training approach that significantly improved mentors’ self-reported skills in addressing work–life issues with their mentees [14].

## Programmatic approaches to design and assess mentee training

Janet Branchaw, PhD, Associate Professor of Kinesiology, School of Education, Faculty Director of the Wisconsin Institute for Science Education and Community Engagement (WISCIENCE), University of Wisconsin-Madison, led a workshop “A Programmatic Approach to Mentoring Early Career Clinical and Translational Researchers.” This workshop focused on how research mentee training programs contribute to building mentorship ecosystems. Participants experienced how backward design can be used to design research training programs. Dr Branchaw shared two researcher development frameworks and assessment tools to monitor trainee development and mentee training curricular activities to support research trainee development [15,16]. Participants explored how to build research training programs that promote shared responsibility for mentoring early career trainees.

Cecilia Patino-Sutton, MD, MEd, PhD, Associate Professor of Population and Public Health Sciences; Director, Workforce Development/Co-Director of the Mentored Career Development in Clinical and Translational Science Award KL2 Program; Keck School of Medicine at the University of Southern California (USC), delivered a talk on “Programmatic Approaches to Incentivize Mentorship.” Dr Patino-Sutton shared a model used for the KL2 Program at USC. The model titled “Predict, Prevent and Overcome” is designed to provide non-directive mentoring interventions to support scholars (e.g., proactively identify needs and problems that the mentoring team or network can fulfill). USC KL2 scholars participate in a 2-week USC Quality by Design (URL: https://sc-ctsi.org/about/initiatives/quality-by-design) training to help them identify barriers in Clinical Research Studies proactively.

## Best practices and strategies for facilitation of mentorship education

Two concurrent workshops were led by Principal Facilitators from the UW-Madison Center for the Improvement of Mentored Experiences in Research (CIMER). Dr Kelly Diggs-Andrews, CEO of Diggs-Andrews Consulting, and Dr Steve Lee, Assistant Dean of Inclusion, Diversity, and Equity at the School of Humanities and Sciences at Stanford University led one workshop “Strategies for Effective Facilitation and Co-facilitation of Mentorship Education.” In the workshop, Drs Lee and Diggs-Andrews articulated the important role that co-facilitation plays in implementing mentor and mentee training. They shared strategies and tools for facilitators to be aware of preferences in communication (e.g., CliftonStrengths Assessment) [17], collaboration (e.g., growth mindset) [18], and conflict management. Participants were introduced to a co-facilitation rubric which can be used to support alignment of expectations between two facilitators. Review of the rubric led to conversations with colleagues to address challenges and support successes in co-facilitation. The presenters also shared examples for facilitators to reflect on and practice DEI values such as seeking increased awareness of their cultural identity and perspective, learn and appreciate their co-facilitator’s contributions, and being thoughtful of co-facilitator’s identities when presenting [19].

The second workshop led by Bruce Birren, PhD, Director of the Genomic Center for Infectious Diseases, Institute Scientist, Broad Institute of MIT and Harvard University and Philip Cheng, PhD, Researcher, Sleep Disorders and Research Center, Henry Ford Health System, Detroit, MI, focused on “Strategies for Addressing Group Dynamics in Mentorship Education Workshops.” Drs Birren and Cheng introduced a general framework “NERVE” (**N**otice, **E**xplore**, R**espond with **V**alidation, **E**scort back on track) for approaching challenging dynamics when facilitating mentorship education. They employed a case-based activity where participants worked together to develop strategies to address group dynamics that might arise when facilitating mentorship training. Guided discussion highlighted the important role that establishing ground rules can play for facilitators in embracing challenging yet critical discussions that arise during mentor and mentee training workshops.

## Promoting a culture of mentorship across diverse institutions and advancing culturally responsive mentorship education

A discussion of “Barriers and Supports for Advancing a Culture of Mentorship Across Diverse Institutions” was led by a panel of distinguished faculty, Roger B. Fillingim, PhD, University of Florida, Winston Thompson, PhD, MS, Morehouse School of Medicine, Leonor Corsino, MD, MHS, Duke University, and moderated by Doris Rubio, PhD, University of Pittsburgh. Panelists led with questions to promote conversations about what may work to foster a culture of mentorship at the institutional level. Examples provided included formation of a Mentor Academy (e.g., Morehouse School of Medicine, University of Florida CTSI) or a centralized office on campus to focus on mentorship and professional development. Challenges to advancing mentorship in varied contexts were shared such as a paucity of mentorship for postdocs, inadequate numbers of research mentors, and establishing mechanisms for mentor accountability. Importantly, strategies were shared to incentivize mentorship (e.g., adding mentorship to faculty promotion criteria) and increase access to effective mentorship (e.g., support from leadership to promote mentorship education).

Richard McGee, PhD, Associate Dean for Professional Development; Professor of Medical Education, Feinberg School of Medicine; Northwestern University, led a session titled, “Culturally Aware Mentorship Training” to introduce the origins and theoretical underpinnings of the mentor training workshop “Culturally Aware Mentorship” (CAM). CAM was created and tested during the first phase of the National Research Mentoring Network (NRMN) and studied through a second phase NRMN U01 award led by Dr Byars-Winston, titled “Impact of Culturally Aware Mentoring Interventions on Research Mentors and Graduate Training Programs.” Dr McGee shared some reflections on the facilitation of the CAM workshop and participants experienced a component of the CAM workshop by engaging in the sharing of a “Culture Box” where by participants bring an item that reflects their culture and share in a group what the item means to them. This promotes a deeper appreciation of the uniqueness of each person [20].

## Tools for assessment of mentorship education and supporting mentoring relationships

Two workshops focused on tools for assessment of mentorship and for supporting mentoring relationships. One workshop “Tools for Assessing Mentoring Relationships, Mentorship Programs and Mentorship Education” led by Christine Pfund, PhD, Director, Mentorship Initiatives for the Institute for Clinical and Translational Research, Director, CIMER, Principal Investigator, NRMN Coordination Center, and Distinguished Senior Scientist, Wisconsin Center for Education Research at the University of Wisconsin-Madison and Angela Byars-Winston, PhD, Professor of Medicine, Associate Director, ICTR Collaborative Center for Health Equity, Chair, Institute for Diversity Science at the University of Wisconsin-Madison. Drs Pfund and Byars-Winston shared a conceptual framework for understanding the impact of mentorship initiatives. They also shared available tools, including a handout of available instruments for assessing mentorship education efforts, mentorship programs, and mentoring relationships on behalf of their work with Dr Jenna Rogers, Scientist and Director of Research, CIMER at the University of Wisconsin-Madison and other team members. Participants were encouraged to share challenges, gaps in available tools, promising practices, and possible intersections for collaboration.

A parallel workshop “Talking about the Mentoring Relationship: Tools for Supporting Mentors and Trainees in Discussing Relationship Expectations” was led by Melissa McDaniels, PhD, Scientist, Institute for Clinical and Translational Research, Associate Executive Director, CIMER, University of Wisconsin-Madison and Christine A. Sorkness, RPh, PharmD., Distinguished Professor of Pharmacy and Medicine; Senior Associate Executive Director, Institute for Clinical and Translational Research, University of Wisconsin-Madison. In this workshop, the important role of establishing and continually managing expectations between mentors and trainees was discussed. A variety of tools and resources to support the alignment of mentor/mentee expectations in mentoring relationships were shared with participants (e.g., CIMER https://cimerproject.org/online-resources/), which highlight the critical role of expectations and signs of misalignment. Additionally, the session emphasized that mentors play multiple roles and articulated the advantages of mapping a mentoring network.

## Building a national community of practice focused on advancing mentorship

A proposal to launch a CTSA-wide Community of Practice (CoP) to advance mentorship was shared at the conference by a small working group that had convened prior to the conference (Emma Meagher, Doris Rubio, Melissa McDaniels, Christine Pfund, and Pamela Asquith). The proposed mission of the CoP is to convene a community of mentorship experts, practitioners, institutional leaders, and scholars across all career stages who are interested in collaborations to advance the quality of mentorship through education and research across the CTSA consortium and beyond. Conference attendees expressed a high level of interest and enthusiasm to participate in the CoP. In response to a participant interest survey, many offered suggested activities and volunteered to serve in a variety of leadership roles. Since the conference, a Steering and Advisory Committee was formed and the inaugural CoP event “Barriers and Supports to Advancing a Culture of Mentorship” was held on October 17, 2023. This online webinar included a presentation and interactive breakout sessions. More information about the CoP and how to participate is available on this website: https://sites.google.com/wisc.edu/ctsacop

## Conference impact

Session slides, handouts, a participant directory, and resources curated from the meeting were posted to the conference website [https://sites.google.com/wisc.edu/science-of-mentorship/home] and shared with all participants. The newly formed CoP to Advance Mentorship aims to: expand implementation of training modules and new approaches to mentorship education; increase awareness and importance placed by leadership on the creation and implementation of sustainable mentorship education programs; increase interest in building skills in culturally responsive mentorship; increase research collaborations among colleagues, particularly early career scientists; and expand adoption of models for incentivizing and rewarding mentorship quality on a national scale.
